# Extracellular vesicles from oviductal and uterine fluids supplementation in sequential in vitro culture improves bovine embryo quality

**DOI:** 10.1186/s40104-022-00763-7

**Published:** 2022-10-25

**Authors:** Cláudia Lima Verde Leal, Karina Cañón-Beltrán, Yulia N. Cajas, Meriem Hamdi, Aracelli Yaryes, María Gemma Millán de la Blanca, Paula Beltrán-Breña, Rosane Mazzarella, Juliano Coelho da Silveira, Alfonso Gutiérrez-Adán, Encina M González, Dimitrios Rizos

**Affiliations:** 1 Department of Animal Reproduction, National Center Institute for Agriculture and Food Research and Technology (CSIC-INIA), 28040 Madrid, Spain; 2 Departamento de Medicina Veterinária, Faculdade de Zootecnia e Engenharia de Alimentos, Universidade de São Paulo (FZEA-USP), Pirassununga, Brazil; 3 Facultad de Ciencias Agrarias y Ambientales, Programa de Medicina Veterinaria, Fundación Universitaria Juan de Castellanos, Tunja, Colombia; 4 Laboratorio de Biotecnología de la Reproducción Animal, Facultad de Ciencias Agropecuarias, Universidad de Cuenca (UC), EC010205 Cuenca, Ecuador; 5 Department of Anatomy and Embryology, Veterinary Faculty-Universidad Complutense de Madrid (UCM), Madrid, Spain

**Keywords:** Cattle, Cryopreservation, Embryo development, Exosomes, Lipid metabolism, miRNAs, Oviduct, Uterus

## Abstract

**Background:**

In vitro production of bovine embryos is a well-established technology, but the in vitro culture (IVC) system still warrants improvements, especially regarding embryo quality. This study aimed to evaluate the effect of extracellular vesicles (EVs) isolated from oviductal (OF) and uterine fluid (UF) in sequential IVC on the development and quality of bovine embryos. Zygotes were cultured in SOF supplemented with either BSA or EVs-depleted fetal calf serum (dFCS) in the presence (BSA-EV and dFCS-EV) or absence of EVs from OF (D1 to D4) and UF (D5 to D8), mimicking in vivo conditions. EVs from oviducts (early luteal phase) and uterine horns (mid-luteal phase) from slaughtered heifers were isolated by size exclusion chromatography. Blastocyst rate was recorded on days 7–8 and their quality was assessed based on lipid contents, mitochondrial activity and total cell numbers, as well as survival rate after vitrification. Relative mRNA abundance for lipid metabolism-related transcripts and levels of phosphorylated hormone-sensitive lipase (pHSL) proteins were also determined. Additionally, the expression levels of 383 miRNA in OF- and UF-EVs were assessed by qRT-PCR.

**Results:**

Blastocyst yield was lower (*P* < 0.05) in BSA treatments compared with dFCS treatments. Survival rates after vitrification/warming were improved in dFCS-EVs (*P* < 0.05). EVs increased (*P* < 0.05) blastocysts total cell number in dFCS-EV and BSA-EV compared with respective controls (dFCS and BSA), while lipid content was decreased in dFCS-EV (*P* < 0.05) and mitochondrial activity did not change (*P* > 0.05). Lipid metabolism transcripts were affected by EVs and showed interaction with type of protein source in medium (*PPARGC1B*, *LDLR*, *CD36, FASN* and *PNPLA2, P* < 0.05). Levels of pHSL were lower in dFCS (*P <* 0.05). Twenty miRNA were differentially expressed between OF- and UF-EVs and only bta-miR-148b was increased in OF-EVs (*P* < 0.05).

**Conclusions:**

Mimicking physiological conditions using EVs from OF and UF in sequential IVC does not affect embryo development but improves blastocyst quality regarding survival rate after vitrification/warming, total cell number, lipid content, and relative changes in expression of lipid metabolism transcripts and lipase activation. Finally, EVs miRNA contents may contribute to the observed effects.

**Supplementary Information:**

The online version contains supplementary material available at 10.1186/s40104-022-00763-7.

## Introduction

In vitro production (IVP) of bovine embryos is applied to animal breeding programs to enhance reproductive efficiency and genetic gain, and has been widely used commercially [1]. However, improvements are still needed, particularly regarding embryo quality in comparison with in vivo derived (IVD) embryos [2–4]. The lower quality is observed as reduced cryotolerance [5], altered gene expression [3, 6] and metabolism [7, 8], among other factors [9, 10]. Also, lower pregnancy rates are reported [11], which can be related with long-lasting effects of in vitro culture (IVC) [12]. When IVP embryos are exposed to in vivo environment [13] or modifications to IVC conditions are introduced, blastocyst quality can be improved [5], therefore, better IVC systems can be developed to promote embryo quality for higher pregnancy rates and healthy offspring births.

Lower quality and cryotolerance has been associated with increased lipid accumulation in IVP embryos compared with IVD [2, 14], and when lipid contents are reduced by different means, cryotolerance is improved [15–18]. The reasons for lipid accumulation are still unclear, but the use of serum during IVC has been implicated [19–22]. Fetal calf serum (FCS) is a common supplement in IVC, and although it has been related to reduced embryonic quality, it paradoxically stimulates embryo development [20]. Serum-free embryo culture is possible, but altered expression of lipid metabolism genes has been reported [23].

Amidst strategies to improve embryo development in vitro, are efforts to mimic the physiological conditions observed in vivo. Preimplantation embryo development in vivo initiates within the oviduct, where the embryo will remain for about 4 d, before entering the uterus [24]. The embryo will develop mostly dependent on molecules present in oviductal (OF) and uterine fluid (UF), until implantation around day 20 [25]. Components supplied by the female reproductive tract are essential to the developing embryo [26, 27], and are largely absent during IVC. Previous studies have shown that IVC medium conditioned by bovine oviduct epithelial cells (BOECs) [28] or supplemented with low concentrations of OF [29] improve quality and cryotolerance of IVP embryos. Less is known relative to the use of UF, but supplementation of IVC medium with low concentration of OF during the initial 4 d, followed by a sequential culture with a low concentration of UF up to the blastocyst stage also enhanced embryo quality [30].

Extracellular vesicles (EVs) are membranous structures secreted by many cell types and identified in several body fluids. They contain diverse molecules (proteins, lipids, metabolites and nucleic acids, including miRNAs), and are involved in cell signalling in various physiological and pathological processes [31–33]. In the female reproductive system, EVs have been found in several species [34, 35], including in fluids from bovine oviduct [36, 37] and uterus [38, 39]. Supplementation of IVC medium with EVs isolated either from BOECs conditioned medium [28] or from OF [36, 37], showed positive effects on embryo development and quality. EVs isolated from bovine UF also favoured the development and quality of somatic cell nuclear transfer bovine embryos [39]. Additionally, EVs from BOECs conditioned medium had positive effects on embryo cryotolerance, even in the presence of serum [28]. This improvement could be related with lower lipid contents in such embryos, but this parameter was not assessed. Interestingly, Almiñana et al. [37] recently detected several transcripts and microRNAs (miRNAs, small non-coding RNAs acting in post-transcriptional regulation, [40]) related with lipid metabolism in EVs from bovine OF. Therefore, these EVs could potentially modulate embryonic lipid metabolism during culture.

In the present study, we have investigated the effects of EVs from OF and UF, when supplemented to IVC medium in a sequential culture system. We hypothesized that this supplementation could improve embryo development and quality, and affect the lipid contents and expression of lipid metabolism-related genes in IVP embryos. The miRNA contents of these EVs were investigated as well. New knowledge in this field could provide the basis for the development of better culture systems, which could bring improvements to the quality of in vitro produced embryos.

## Materials and methods

### Experimental design

**Fig. 1:**
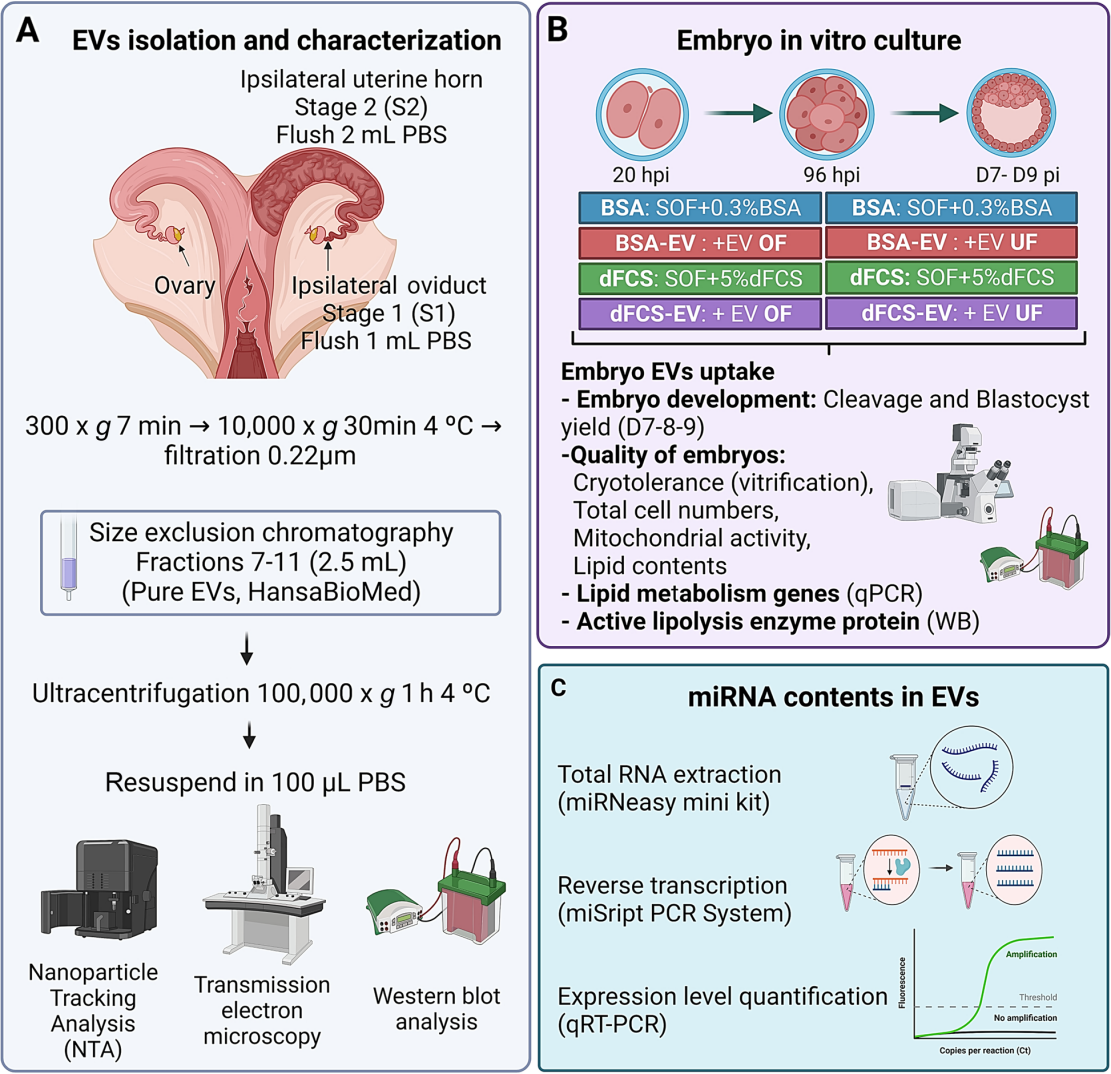
Experimental design. **A** EVs isolation and characterization. Oviducts (*n* = 5) and uterine horns (*n* = 5), ipsilateral to a Stage 1 (oviduct) or Stage 2 (uterus) corpus luteum, were flushed with PBS (1 and 2 mL, respectively), and after centrifugation to remove cells and cellular debris, samples were filtered (0.22 μm) and EVs isolated by size exclusion chromatography. EVs samples were concentrated by ultracentrifugation and pellets resuspended in 100 μL PBS. Thirty microliters from each of the five EVs suspensions from oviduct (150 μL) and uterus (150 μL) were pooled and from this, 5 μL were used for EVs characterization by nanoparticle tracking analysis (NTA) and 5 μL for transmission electron microscopy (TEM), and the rest was frozen at − 20 °C and used in IVC. The remaining volume of the original five EVs samples (70 μL) was used for miRNA analysis. To have enough protein amount (35 μg) for EV biomarkers to be detectable by western blot, a pool of 10 oviducts (Stage 1) and 10 uterine horns (Stage 2) flushings was obtained and prepared the same way. **B** Embryo culture in vitro. At approximately 20 h after insemination, presumptive zygotes were cultured in 4 treatments: BSA: SOF with 0.3% BSA (3 mg/mL, w/v); dFCS: SOF with 5% dFCS; BSA-EV: SOF with 0.3% BSA supplemented with 3 × 10^5^ EVs/mL from OF (D1 to D4) and 3 × 10^5^ EVs/mL from UF (D4 to D9); and dFCS-EV: SOF with 5% dFCS supplemented with 3 × 10^5^ EVs/mL from OF (D1 to D4) and 3 × 10^5^ EVs/mL from UF (D4 to D9). BSA and dFCS treatments underwent media renewal at 96 hpi (day 4). Blastocyst development was assessed on days 7, 8 and 9. A representative number of day 7–8 blastocysts from each group was assessed for quality either by vitrification/warming (survival rate at 24, 48 and 72 h) or fixed and stained for total cell number, mitochondrial activity and lipid content and analysis. In addition, day 7–8 blastocysts were frozen in liquid nitrogen in groups of 10 and stored at − 80 °C for gene expression or western blot for protein analyses. **C** miRNA contents in EVs. Total RNA in oviducts and uteri EVs samples (*n* = 3) was extracted with a miRNeasy mini kit, reversed transcribed with the miScript PCR System and relative miRNA levels determined by qRT-PCR

The developmental capacity of bovine zygotes and the quality of the produced blastocysts cultured in vitro with EVs from OF and UF, mimicking the physiological preimplantation environment in vivo was assessed. At approximately 20 h after insemination, presumptive zygotes were cultured in 4 treatments: (i) BSA: synthetic oviductal fluid (SOF) with 3 mg/mL BSA; (ii) dFCS: SOF with 5% dFCS; (iii) BSA-EV: SOF with 3 mg/mL BSA supplemented with 3 × 10^5^ EVs/mL from OF (D1 to D4) and 3 × 10^5^ EVs/mL from UF (D4 to D9); and (iv) dFCS-EV: SOF with 5% dFCS supplemented with 3 × 10^5^ EVs/mL from OF (D1 to D4) and 3 × 10^5^ EVs/mL from UF (D4 to D9). BSA and dFCS treatments underwent media renewal on day 4 (Fig. 1). Blastocyst development was assessed on days 7, 8 and 9. To assess blastocyst quality a representative number of day 7–8 blastocysts from each treatment was either vitrified/warmed, and survival rate was recorded every 24 h up to 72 h after warming, or fixed and stained for mitochondrial activity, lipid content and total cell number analysis. In addition, day 7–8 blastocysts were frozen in liquid nitrogen and stored at − 80 °C for gene expression or western blot for protein analyses. miRNA contents in EVs were also analysed.

### Materials

Unless otherwise stated, all chemicals were purchased from Sigma-Aldrich Corporation (St Louis, MO, USA).

### EVs depletion of FCS for use in IVC experiments

To produce foetal calf serum (FCS) depleted from its EVs (dFCS), heat-inactivated FCS was ultra-centrifuged at 100,000 × *g* for 18 h at 4 °C using an Optima-L-100XP Beckman Coulter ultracentrifuge. Under a laminar hood the supernatant (dFCS) was collected, aliquoted and stored at − 20 °C for later use in embryo culture. The same batch was used in the entire experiment.

### Isolation of extracellular vesicles (EVs)

Isolation and characterization of EVs from bovine oviduct and uterine fluids analysis was performed according to Cañón-Beltrán et al. [41], with minor modifications, as described below.

#### Oviductal and uterine flushing for EVs isolation

Five oviducts and five uteri from slaughtered heifers were selected according to the stage of the corpus luteum, and transported to the laboratory on ice. Oviducts corresponding to Stage 1 (from day 1 to day 4 of the estrous cycle) and uteri to Stage 2 (from day 5 to day 10 [42];) ipsilateral to the corpus luteum were each trimmed free of associated tissues and washed in cold Ca^2+^ and Mg^2+^-free phosphate-buffered saline (PBS^−^). Each oviduct was flushed (1 mL cold PBS^−^) from ampulla to isthmus while each uterine horn was flushed (2.2 mL cold PBS^−^) towards the uterine tubal junction. All manipulations were performed at 4 °C. To remove cells and cellular debris, each oviduct and uterine flush sample was centrifuged once at 300 × *g* for 7 min, then the supernatant was centrifuged again at 10,000 × *g* for 30 min at 4 °C [43]. The resulting supernatants were each separately passed through a 0.22-μm filter and stored at 4 °C until EVs isolation the next day.

#### Extracellular vesicle isolation by size exclusion chromatography

OF-EVs and UF-EVs were isolated by size exclusion chromatography (SEC), according to manufacturer’s instructions (Pure EVs®, HBM-PEV; Hansa BioMed Life Sciences). Briefly, columns were washed with 30 mL PBS^−^ and then each OF (≈ 1 mL) or UF (≈ 2 mL) sample was loaded onto the top of a SEC column, and when the sample was completely within the column, 11 mL PBS^−^ were loaded. The first 3.0 mL were discarded and the following 2.0 mL of EVs-rich fractions were collected. Next, to concentrate the 2.0 mL EVs samples, they were submitted to ultracentrifugation (UC) at 100,000 × *g* for 1 h at 4 °C (Optima L-90 K ultracentrifuge with a swinging-bucket rotor SW 41 Ti, Beckman Coulter, Fullerton, CA, USA). Supernatants were discarded and each pellet was suspended in 100 μL cold PBS. Thirty microliters were taken from each of the five EVs suspensions of OF and UF and pooled (150 μL final pool volume), and stored at 4 °C. From each pool, 5 μL were used for characterization by nanoparticle tracking analysis (NTA) and another 5 μL for transmission electron microscopy (TEM). The remaining 70 μL volume of each of the original five EVs samples (Stage 1 OF and Stage 2 UF), were separately stored at − 80 °C until used for miRNA analysis. In order to obtain sufficient protein for western blot analysis, another ten oviducts and uteri were collected to obtain EVs from Stage 1 OF and from Stage 2 UF, which were isolated in the same way and the EVs suspensions were entirely used for the western blots.

### EVs characterization

#### Nanoparticle tracking analysis (NTA)

Analysis of concentration and size distribution of EVs was performed using a NanoSight LM-10 system equipped with a CCD video camera and particle-tracking software NTA 3.1 Build 3.1.45 (NanoSight Ltd., Minton Park, UK). Five microliters of EVs solution were diluted in 95 μL of filtered PBS^−^. The NTA measurement conditions were detection threshold 2 to 3, camera level 13, temperature 22 °C and measurement time 60 s. Three recordings were performed for each sample. After EVs concentration of each sample was determined, the stored pooled samples were diluted to a standardized concentration (3 × 10^5^ particles/mL, [28, 36]), aliquoted and frozen at − 20 °C for later use in embryo culture experiments.

#### Transmission electron microscopy (TEM)

Five microliters of each EVs suspension were diluted in 45 μL, and then two 25-μL droplets of EVs preparations were allowed to adsorb onto formvar/carbon-coated 200 mesh copper grids (Agar Scientific, Essex, UK) for 1 min at room temperature. Grids were then washed twice with distilled water. For negative staining, grids were transferred to a 50-μL droplet of 2% uranyl acetate for 20 s, followed by blotting the excess liquid, and air-dried. TEM visualizations were performed at the National Center for Electron Microscopy of the Universidad Complutense de Madrid (ICTS-CNME-UCM) using a JEOL JEM1010 (100 kV) transmission electron microscope (Jeol Ltd., Tokyo, Japan) equipped with a Megaview II CCD camera integrated into iTEM Olympus Soft Imaging Solutions software (Olympus, Tokyo, Japan).

#### Western blot for EV marker proteins

EVs preparations were lysed in 1× RIPA buffer (Cell Signaling Technology, 9806S), supplemented with 1× protease, phosphatase Inhibitor Cocktail (Roche, Basel, Switzerland), and protein concentrations determined by the bicinchoninic acid assay (BCA) (Micro BCA Protein Assay Kit; 23325). A total of 35 μg of protein per sample was suspended in Laemmli loading buffer, then loaded and separated in a 4%–12% gradient SDS-PAGE polyacrylamide gel. Proteins were transferred onto nitrocellulose membranes (GE Healthcare Life Sciences Whatman™). The membranes were washed in distilled water and blocked with 3% bovine serum albumin (BSA) in PBS-T (PBS + 0.1% Tween-20) for 30 min at room temperature. The membranes were incubated with primary antibodies diluted in PBS-T with 3% BSA overnight at 4 °C with gentle shaking. Three primary antibodies were used as EVs biomarkers: anti-tetraspanin cell surface protein CD9 antigen (1:1000, anti-CD9 mAb, 13403, Cell Signaling Technology, D3H4P, Danvers, MA, USA), anti-heat shock protein 70 (1:1000, anti-HSP70 mAb, C92F3A-5, Enzo Life Sciences, NY, USA); anti-ALG-2-interacting protein X protein (1:1000, anti-ALIX mAB, sc-53,540, Santa Cruz Biotechnology, CA, USA). As negative control, anti-Calnexin (1:1000, anti-CANX mAb sc-23954, Santa Cruz Biotechnology, CA, USA) was used to indicate the absence of cell contamination in EVs samples. Next, the membranes were washed with PBS-T and incubated for 2 h under agitation with horseradish peroxidase (HRP) conjugated secondary antibodies: goat anti-rabbit IgG-HRP (1:5000, 7074S, Cell Signaling Technology, Danvers, MA, USA) or horse anti-mouse IgG-HRP (1:5000, 7076S, Cell Signaling Technology, Danvers, MA, USA). The membranes were again washed three times in PBS-T for 5 min and incubated for 1 min in the Immobilon Forte Western HRP substrate (#WBLUF0100, Millipore, Burlington, MA, USA) and revealed by chemiluminescence with an Image Quant LAS500 biomolecular imager (GE Healthcare Life Sciences, USA, 29005063). Blood, pancreas, lungs and ureters tissues of mice were used as positive controls for CD9, HSP70, ALIX and CANX, respectively.

### EVs miRNA contents analysis

#### EVs RNA extraction

Total RNA (including small RNAs) was extracted (*n* = 3 per group) using the miRNeasy Mini Kit (217004, Qiagen, Venlo & Limburg, the Netherlands) according to manufacturer’s instructions. RNA concentration and quality were evaluated by NanoDrop™ (Thermo Scientific) and samples with the highest RNA amount were used for analysis.

#### miRNAs analysis

Analysis of miRNA contents was performed as described by Da Silveira et al. [44], with minimal modifications. Briefly, reverse transcription was conducted on the total RNA (120 ng/sample) using the miScript PCR System (Qiagen, Venlo & Limburg, the Netherlands) according to the manufacturer’s instructions. Total RNA, including small RNA fraction, were incubated with 5 × miScript Hiflex Buffer, 10 × miScript Nucleic mix, RNAse free water, and miScript reverse transcriptase at 37 °C for 60 min, followed by 5 min at 95 °C. The relative abundance levels of 383 mature miRNAs were established via quantitative real-time (qRT-PCR), using the miSCRIPT II RT kit (Qiagen, Venlo & Limburg, the Netherlands), according to manufacturer’s instructions. Reactions were prepared in 6 μL master mix containing 2 × QuantiTect SYBER Green PCR Master Mix (Qiagen), 10 × miScript Universal Primer, miRNA specific forward primers and 0.024 μL of 1:4 diluted cDNA. qRT–PCR was conducted in a 384-well plate using the Quantstudio 6 System (Applied Biosystems). The PCR cycling conditions were 95 °C for 15 min, 45 cycles of 94 °C for 10 s, 55 °C for 30 s, and 70 °C for 30 s followed by a melting curve analysis to confirm the specificity of cDNA amplification. To quantify miRNAs expression level in EVs isolated from OF and UF, raw cycle threshold (Ct) values were normalized to the geometric mean of bta-miR-99b, Hm/Ms/Rt T1 snRNA and RNT43 snoRNA, as internal controls. OF and UF-EVs samples were analysed in triplicate, and only miRNAs with a Ct value less than 37, and detected in at least two of the three samples in each group, were considered to be present.

### In vitro embryo production

#### Oocyte collection and in vitro maturation

Immature cumulus-oocyte complexes (COCs) were obtained by aspirating follicles (2–8 mm) from the ovaries of mature heifers and cows collected from local abattoirs. COCs were selected and matured in four-well dishes (Nunc, Roskilde, Denmark) in 500 μL TCM-199 medium supplemented with 10% (v/v) FCS and 10 ng/mL epidermal growth factor (EGF), in groups of 50 COCs per well, for 24 h at 38.5 °C and under an atmosphere of 5% CO_2_ in air with maximum humidity.

#### Sperm preparation and in vitro fertilization (IVF)

IVF was performed as described previously [36]. Briefly, frozen semen straws (0.25 mL) from an Asturian Valley bull previously tested for IVF were thawed at 37 °C in a water bath for 1 min and sperm cells were selected on a gradient of Bovipure (Nidacon Laboratories AB, Göthenborg, Sweden). Sperm concentration was determined and adjusted to a final concentration of 1 × 10^6^ sperm cells/mL. Gametes were coincubated for 18–22 h in 500 μL fertilization medium (Tyrode’s medium with 25 mmol/L bicarbonate, 22 mmol/L sodium lactate, 1 mmol/L sodium pyruvate and 6 mg/mL fatty acid-free BSA) supplemented with 10 μg/mL heparin sodium salt (Calbiochem, San Diego, CA, USA) in a four-well dish, in groups of 50 COCs per well, under an atmosphere of 5% CO_2_ with maximum humidity at 38.5 °C.

#### In vitro culture of presumptive zygotes

At approximately 18–22 h post-insemination (hpi), presumptive zygotes were denuded of cumulus cells by vortexing for 3 min and then cultured in groups of 20-25 zygotes in 25 μL droplets of culture medium (synthetic oviductal fluid, (SOF) [45]; with 4.2 mmol/L sodium lactate, 0.73 mmol/L sodium pyruvate, 30 μL/mL basal medium Eagle (BME) amino acids, 10 μL/mL minimum essential medium (MEM) amino acids and 1 μg/mL phenol-red). Culture medium was supplemented with 3 mg/mL BSA or 5% dFCS in the presence (BSA-EV and dFCS-EV) or absence (BSA and dFCS) of 3 × 10^5^ EVs/mL from OF (D1 to D4) and UF (D5 to D9), under mineral oil at 38.5 °C and an atmosphere of 5% CO_2_, 5% O_2_ and 90% N_2_. BSA was included as an additional comparison group as it has been shown to improve embryo quality and cryotolerance [30, 36].

### Assessment of embryo EVs uptake, development and quality

#### EVs uptake by embryos

EVs isolated from OF and UF were labelled with a lipophilic green fluorescent dye (PKH67, Sigma, USA) as described by Almiñana et al. [37] with some modifications. Firstly, 25 μL of the EVs suspension in PBS were mixed with 125 μL of diluent C (Cell mixture) and for the negative control, 25 μL of PBS^−^ without EVs were also mixed with 125 μL of diluent C. Next, the dye was diluted in diluent C (1:250), and each sample (EVs and controls) was then placed into 125 μL of the dye mix and incubated for 5 min at room temperature (final concentration of dye is 5 × 10^−6^ mol/L). To stop the labelling reaction 250 μL of 1% BSA in PBS^−^ was added per sample for 1 min. The samples (dye-EVs and dye-PBS^−^ negative control) were washed four times (three 15 min centrifugations at 2000 × *g* and one for 20 min) with 9 mL PBS^−^ in Amicon Vivaspin® filters (Sartrius Stedim, Germany). The samples (100 μL) were then filtered with a 0.22-μm filter and diluted 1:1 with SOF medium (2 ×) without phenol red and with BSA at 3 mg/mL (final concentration). Embryos were cultured for 5 h in this medium. The labelled EVs from OF and the PBS^−^ negative control (labelled but without EVs) were used in the culture of D3 8-cell embryos, and the labelled EVs from UF and its PBS^−^ negative control were used in the culture of D8 blastocysts. Embryos were then collected and washed twice in PBS to remove any labelled vesicles that were not internalized, followed by fixation in 4% paraformaldehyde (PF) during 30 min, washing, and labelling with Hoechst 33342. Finally, embryos (10–14 per group) were placed in a droplet of ProLong Diamond Antifade Mountant on a glass slide, covered with a coverslip, and then analysed under a multispectral confocal microscope (Leica TCS-SP8 STED 3X) using the 63 × objective.

#### Embryo development

Cleavage rate was recorded on day 2 (48 hpi) and cumulative blastocyst yield was recorded on days 7, 8 and 9 pi. Throughout the study, twenty-eight embryo production runs were performed (presumptive zygotes cultured: BSA = 1584, BSA-EVs = 1853, dFCS = 1594 and dFCS+EVs = 1473) to generate blastocysts for development rates determination and for the quality and expression analyses.

#### Embryo quality

##### Blastocyst vitrification

The ability of the blastocyst to withstand cryopreservation was used as an indicator of quality. Day 7–8 blastocysts from each treatment (*n* = 67, 52, 86 and 64 for BSA, BSA-EV, dFCS and dFCS-EV, respectively) were vitrified in holding medium (HM), consisting of TCM 199 supplemented with 20% (v/v) FCS, and cryoprotectants as described previously [2] in a two-step protocol using the Cryoloop device (Hampton Research, Aliso Viejo, CA, USA). In the first step, HM was supplemented with 7.5% ethylene glycol and 7.5% dimethyl sulfoxide; in the second step, HM was supplemented with 16.5% ethylene glycol, 16.5% dimethyl sulfoxide and 0.5 mol/L sucrose. Blastocysts were warmed in two steps in HM with 0.25 and 0.15 mol/L sucrose and then cultured in 25-μL droplets of SOF with 5% FCS. Survival was defined as re-expansion of the blastocoel and its maintenance for 72 h after warming.

##### Mitochondrial activity measurement, lipid content quantification and total cell number in blastocysts

Mitochondrial activity, lipid contents and total cell number in blastocysts were used as additional parameters of embryo quality. For mitochondrial activity, blastocysts (D7–8) from each treatment (*n* = 29, 29, 33 and 27 for BSA, BSA-EV, dFCS and dFCS-EV, respectively) were suspended in 100 μL PBS without calcium and magnesium supplemented with 0.1% polyvinylpyrrolidone (PVP). Next, they were equilibrated for 15 min in culture medium supplemented with 5% FCS and then incubated for 30 min at 38.5 °C in 400 nmol/mL MitoTracker DeepRed (Molecular Probes, Eugene, USA); blastocysts were then fixed in 4% PF for 30 min at room temperature. For lipid content analysis (*n* = 28, 26, 25 and 29 for BSA, BSA-EV, dFCS and dFCS-EV, respectively), the blastocysts were fixed in PF and then permeabilized with 0.1% saponin for 30 min and stained for 1 h with 20 μg/mL Bodipy 493/503. For total cell number the blastocysts (*n* = 57, 55, 58 and 56 for BSA, BSA-EV, dFCS and dFCS-EV, respectively) were stained with Hoëchst 33,342 (10 μg/mL) for 30 min, after being stained either for mitochondria or for lipids. After each stain, the blastocysts were washed in PBS + 0.1% PVP three times for 5 min each. Finally, blastocysts were mounted in 3.8 μL mounting medium (ProLong Gold, Thermo Fisher Scientific) between a coverslip and a glass slide and sealed with nail polish.

Slides were examined using a laser-scanning confocal microscope (Leica TCS SP2) equipped with an argon laser excited at 488 nm and whose emission spectrum is 500–537 nm for visualization of lipid droplets. For mitochondria, excitation and emission were set at 543 nm and 580–650 nm, respectively. The format, laser, gain and offset were kept constant for every sample.

For the assessment of mitochondrial activity, the fluorescence signal intensity (pixels) was quantified. Serial sections of 5 μm were made for each blastocyst and a maximum projection was accomplished for each one. Images obtained were evaluated using the ImageJ program (NIH; http://rsb.info.nih.gov/ij/). After selection using the freehand selection tool, each blastocyst was measured to determine its area and its integrated density (IntDen), which corresponds to pixel intensity. In addition, the background fluorescence of an area outside the blastocyst was measured. Fluorescence intensity in each blastocyst was determined using the following formula: Relative fluorescence = IntDen – (area of selected blastocyst × mean fluorescence of background readings). Fluorescence intensities are expressed in arbitrary units (a.u.) [46].

The lipid quantity in blastocysts was obtained by analysis of the total area of lipids in each embryo. We captured three images of each blastocyst: one in the middle of the blastocyst (the image with the largest diameter) and the other two in the middle of the resulting halves. We used a 63 × objective at a resolution of 1024 × 1024 and images were analysed using the ‘nucleus counter’ tool, set to detect, distinguish and quantify droplet areas with the ImageJ software (NIH, USA) and expressed as the area of lipid droplets relative to the total area of the blastocyst without its cavity (μm^2^). For blastocysts, lipid quantity was corrected by area, to account for varying blastocyst sizes. After verification of a significant correlation (*r*^2^ = 0.84 and *P* < 0.0001 by Pearson’s correlation test) between lipid quantity of three sections in 30 blastocysts (10 per group) we chose the section with the largest area per embryo to be analysed [22]. The number of cells per blastocyst was determined by counting the Hoechst-stained cells under an epifluorescence microscope (Nikon 141,731) equipped with a fluorescent lamp (Nikon HB-10104AF) and UV-1 filter.

### Gene expression analysis

Gene expression analysis was performed using day 7–8 blastocysts (pools of 10 in three replicates per treatment). All samples were washed in PBS, snap-frozen in LN_2_ and stored at − 80 °C until mRNA extraction analyses. Poly(A) RNA was extracted using the Dynabeads mRNA Direct Extraction Kit (Ambion; Thermo Fisher Scientific) with minor modifications [47]. Immediately after poly(A) RNA extraction, reverse transcription (RT) was performed using a MMLV Reverse Transcriptase 1st-Strand cDNA Synthesis Kit according to the manufacturer’s instructions (Epicentre Technologies). Poly(T) random primers and Moloney murine leukaemia virus (MMLV) high-performance reverse transcriptase enzyme were used in a total volume of 40 μL to prime the RT reaction and to produce cDNA. Tubes were heated to 70 °C for 5 min to denature the secondary RNA structure and the RT mix was then completed by adding 50 units of reverse transcriptase. Samples were incubated at 25 °C for 10 min, to help the annealing of random primers, followed by incubation at 37 °C for 60 min, to allow the RT of RNA, and finally at 85 °C for 5 min to denature the enzyme. All mRNA transcripts were quantified in duplicate using a Rotorgene 6000 Real Time Cycler (Corbett Research). RT–quantitative polymerase chain reaction (qPCR) was performed by adding 2 μL aliquot of each cDNA sample (〜60 ng/μL) to the PCR mix (GoTaq qPCR Master Mix, Promega) containing the specific primers to amplify transcripts for the genes (Additional file 1: Table S1). The selection of genes to be evaluated was carried out considering the expression of key genes in lipid metabolism. All primers were designed using Primer-BLAST software (http://www.ncbi.nlm.nih.gov/tools/primer-blast/) to span exon–exon boundaries when possible. For quantification, RT-qPCR was performed as described previously [48]. The PCR conditions were tested to achieve efficiencies close to 1. Relative expression levels were quantified by the comparative cycle threshold (Ct) method [49]. Values were normalized using two housekeeping (HK) genes: *H2AZ1* and *ACTB* (Additional file 1: Table S1). Fluorescence was acquired in each cycle to determine the threshold cycle or the cycle during the log-linear phase of the reaction at which fluorescence increased above the background for each sample. Within this region of the amplification curve, a difference of one cycle is equivalent to a doubling of the amplified PCR product. According to the comparative Ct method, the ΔCt value was determined by subtracting the mean Ct value of the two HK genes from the Ct value of the gene of interest in the same sample. The calculation of ΔΔCt involved using the highest treatment ΔCt value (i.e. the treatment with the lowest target expression) as an arbitrary constant to subtract from all other ΔCt sample values. Fold-changes in the relative gene expression of the target were determined using the formula 2^-ΔΔCt^.

### Protein analysis in blastocysts

Protein analysis was performed using day 7–8 blastocysts (pools of 20 in 3 replicates per treatment). Embryo samples were lysed in RIPA buffer supplemented with 1 × protease, phosphatase Inhibitor Cocktail. Proteins were resolved in SDS-PAGE (4%–12% acrylamide gel loading 45 μL of total protein per well) and transferred to a nitrocellulose membrane (Amersham™ Protran™ 0.45 NC), blocked with 3% BSA in PBS-T and incubated overnight at 4 °C with the following primary antibodies: HSL rabbit (1:1000 in 3% BSA + 0.1% PBS-T, #4107S, Cell Signaling Technology, Boston, MA, USA) and pHSL rabbit (phospho-HSL, 1:1000 in 3% BSA + 0.1% PBS-T, #4139S, Cell Signaling Technology, Boston, MA, USA). The membranes were washed three times in 1 × TBS-T for 5 min and incubated for 2 h at room temperature with a goat anti-rabbit IgG-HRP (1:5000 in 1% BSA + 0.1% PBS-T, #7074, Cell Signaling Technology, Danvers, MA, USA). The membranes were again washed three times in 1 × TBS-T for 5 min and incubated for 5 min in Enhanced Chemiluminescence kit (RPN2109, ECL™, Amersham GE Healthcare, Buckinghamshire, UK) and detected by an ImageQuant LAS 500 chemiluminescence CCD camera (GE Healthcare Life Sciences, USA, 29005063).

The monoclonal anti-β-actin−peroxidase antibody produced in mouse (Merk, Darmstadt, Germany, A3854) was used as the loading control. For this purpose, after the chemiluminescent detection of HSL and pHSL, membranes were stripped and then wash extensively in TBS-T and repeating the blocking step, the membrane is re-probed with an anti-actin antibody. In all cases, intensities of protein bands [(optical density (OD)] were quantified by ImageJ software and normalized relative to the abundance of actin in each lane and phosphorylation level was expressed as phosphorylated pHSL/HSL. The ratio of the OD of the protein concerned (HSL/pHSL) in relation to actin is presented in the form of bar charts [41].

### Statistical analysis

Cleavage and blastocyst rates, and vitrification data were analysed by Chi-square test. Number of cells per blastocyst, mitochondrial activity, lipid content, relative mRNA abundance and protein level (pHSL/HSL) data were submitted to two-way analysis of variance (ANOVA) followed by Tukey’s test to determine differences between treatments. The association analysis for lipid quantification in blastocyst was determined by linear regression *r*^2^ test and significance of the correlation by *F* test. Values were considered significantly different at *P* < 0.05. Unless otherwise indicated, data are presented as the mean ± SEM. All analyses were made with the SigmaStat software package (Jandel Scientific, San Rafael, CA, USA). For miRNA, data were displayed as the mean ± SD of replicate samples. Statistical analyses were performed using SAS 9.3 Software (SAS Institute). A Student’s *t*-test was used to assess statistical differences, and normality was verified using a Shapiro–Wilk test, where a statistical difference of 5% was considered to be significant.

## Results

### OF and UF EVs characterization

#### NTA

**Fig. 2:**
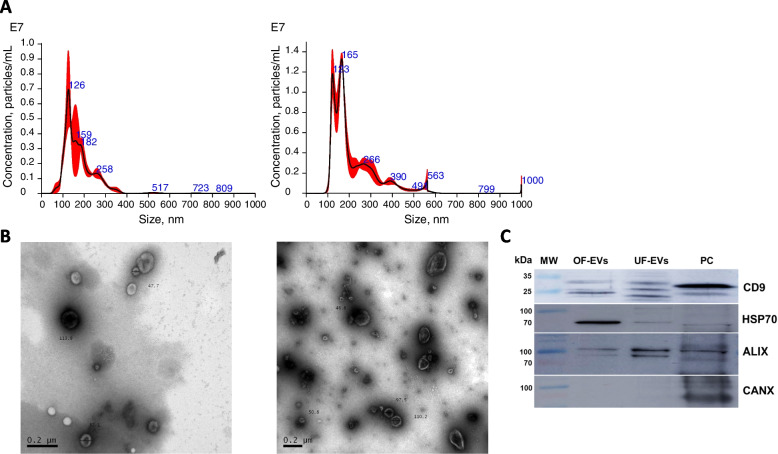
OF and UF-EVs characterization. **A** Averaged finite track length adjustment Concentration/Size graphs for NTA of particles in fluid isolated from oviducts (left) and uterine horns (right). **B** Representative images from electron microscopy analyses of OF (left) and UF (right) samples. Some EVs and their sizes are indicated in the images. **C** Representative images of membranes treated for detection of EVs marker-proteins (CD9, HSP70 and ALIX) and EVs negative protein (CANX) in samples isolated from oviduct (OF-EVs) and uterus (UF-EVs). PC = positive controls (CD9 = blood, HSP70 = pancreas, ALIX = lung, CANX = urethra). MW = molecular weight marker. Numbers indicate the MW. Expected MW: CD9 = 22, 24 and 35 kDa, HSP70 = ~ 70 kDa, ALIX = 90 kDa and CANX = 90 kDa

This analysis was performed to characterize the size and concentration of particles present in the pools of the five samples isolated from OF and UF. NTA showed that concentration in OF was 2.97 × 10^10^ particles/mL, while the mean size was 177.5 nm and modal size 137.2 nm. In UF concentration was 7.98 × 10^10^ particles/mL and mean and modal sizes were 216.5 and 151.2, respectively. Sizes were within the expected range for the EVs being studied. Concentration/Size distribution of EVs from oviductal and uterine samples, which were used for IVC experiments, are shown in Fig. 2A.

#### TEM

Figure 2B shows representative images from TEM analysis for the presence and morphology of EVs in oviductal and uterine samples. The structures observed are compatible with EVs, confirming NTA data and their presence in the samples analysed.

#### Western blotting

Marker proteins for EVs CD9, HSP70 and ALIX were detected in OF and UF samples (Fig. 2C). The negative control CANX was not detected in any samples, except for its positive control tissue (urethra). These observations further confirm the presence of EVs in the samples analysed and the absence of cell contamination.

### In vitro culture

#### EVs uptake

**Fig. 3:**
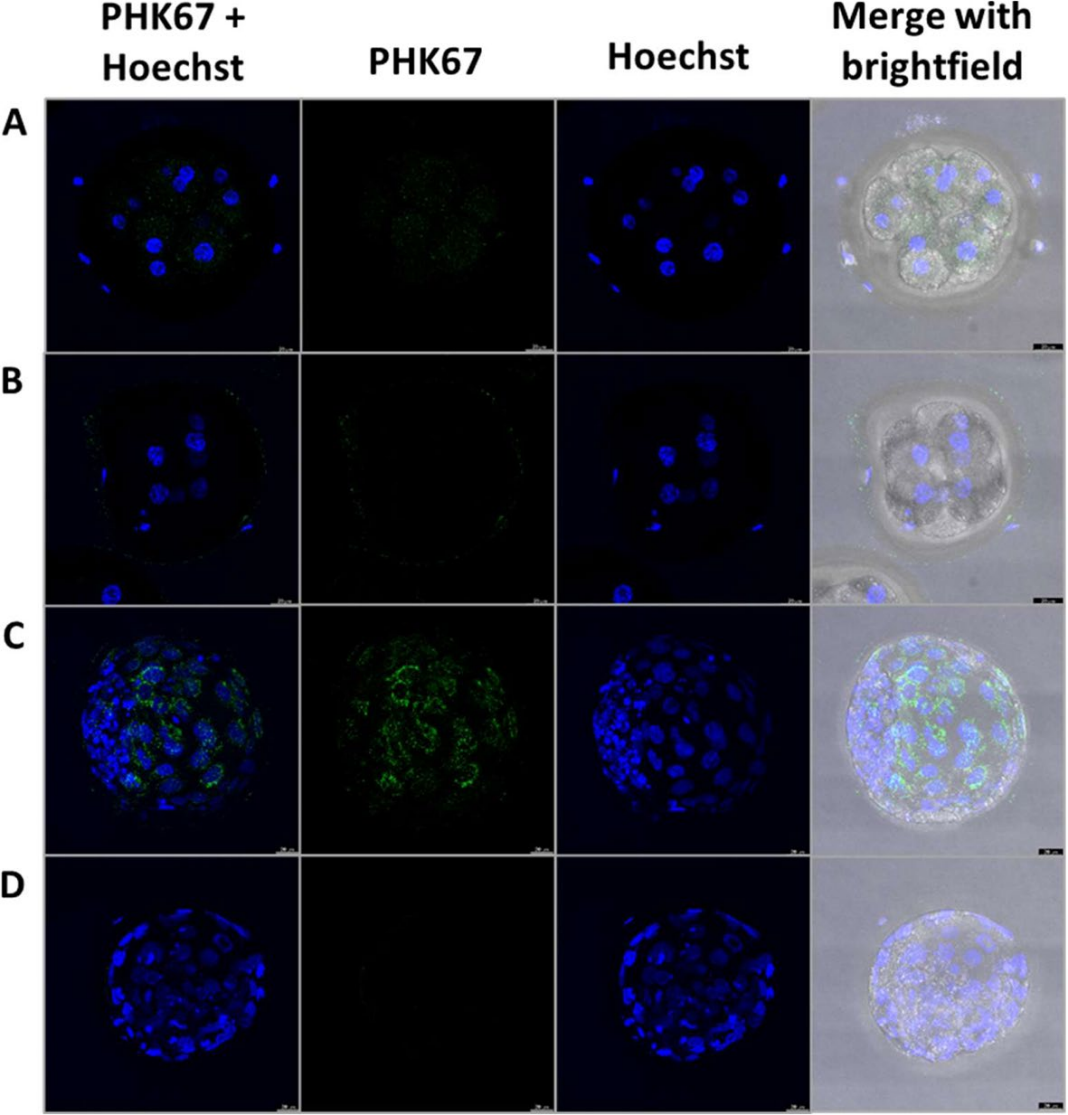
Representative image of embryo on day 3 of IVC with EVs from OF and UF. **A** Representative image of embryo on day 3 of culture in vitro with EVs from OF and **C** on day 8 with EVs from UF. Nuclei in blue indicate cells (blastomeres) and EVs labelled with PHK67 (green). **B** Negative control for OF in D3 embryo and **D** for UF in D8 embryo. 63 ×

In order to confirm that EVs added to the media were taken up by embryos and, therefore, potentially capable of affecting their development and/or quality, EVs from OF and UF were fluorescently labelled and then supplemented to embryo culture. After 5 h of culture of 8-cell embryos (day 3) and blastocysts (day 8) with the OF and UF labelled EVs, respectively, EVs uptake by embryos was confirmed. As observed in Fig. 3, punctuated fluorescence was detected inside the blastomeres of the 8-cell embryos and blastocysts.

#### Embryo development

As shown in Table 1, cleavage was not affected by treatments (*P* > 0.05) and the mean rate was 89.9%. Blastocyst rate on day 7 of culture, however, showed that treatments with BSA, with or without EVs (16.2% and 14.1%, respectively, *P* > 0.05), were lower (*P* < 0.05) than dFCS treatments, with or without EVs (30.5% and 31.1%, respectively, *P* > 0.05). On D8, BSA and BSA-EV (31.0% and 26.2%, respectively) still had lower (*P* < 0.05) blastocyst rates than dFCS and dFCS-EV (40.7% and 39.8%, respectively, *P* > 0.05), and BSA-EVs had lower development than BSA (*P* < 0.05). The same pattern was seen on D9, where BSA and BSA-EV (34.6% and 29.2%, respectively), were lower (*P* < 0.05) than dFCS and dFCS-EV (41.7% and 41.4%, respectively, *P* > 0.05) and BSA-EV was lower than BSA (*P* < 0.05).

**Table 1:** Cleavage (D2) and cumulative blastocyst development rates (D7–9) after in vitro culture with BSA or dFCS supplemented or not with OF-EVs (D1–4) and UF-EVs (D5–8) (28 replicates)

Treatment	IVC ***n***	Cleaved	Blastocysts		
		D2	D7	D8	D9
		***n*** (% ± SEM)	***n*** (% ± SEM)	***n*** (% ± SEM)	***n*** (% ± SEM)
BSA	1584	1427 (89.5 ± 1.0)	248 (16.2 ± 1.5)^a^	484 (31.0 ± 1.9)^a^	548 (34.6 ± 1.7)^a^
BSA-EV	1853	1677 (89.7 ± 1.1)	261 (14.1 ± 1.7)^a^	496 (26.2 ± 2.0)^b^	555 (29.2 ± 2.1)^b^
dFCS	1594	1435 (89.5 ± 0.9)	476 (30.5 ± 2.0)^b^	648 (40.7 ± 2.5)^c^	659 (41.7 ± 2.4)^c^
dFCS-EV	1473	1337 (90.7 ± 0.9)	460 (31.1 ± 2.5)^b^	590 (39.8 ± 2.7)^c^	615 (41.4 ± 2.8)^c^

^a,b,c^Values with different superscripts within a column are significantly different *(P* < 0.05)

### Embryo quality

#### Vitrification

As a parameter to assess the quality of embryos produced in the different treatments, the survival rate after vitrification and warming of blastocysts cultured up to 72 h in SOF with 5% dFCS was measured (Table 2). Survival rate at 72 h was highest for dFCS-EV (87.8%, *P* < 0.05) compared with its respective control (dFCS = 69.2%) and the other treatments, which were similar among themselves. All treatments had high survival at this time point ranging from 67.1% to 73.1% (*P* > 0.05).

**Table 2:** Survival rates after vitrification and warming of day 7–8 blastocysts cultured with BSA or dFCS supplemented or not with OF-EVs (D1–4) and UF-EVs (D5–8)

Treatment	IVC ***n***	4 h	24 h	48 h	72 h
		***n*** (% ± SEM)	***n*** (% ± SEM)	***n*** (% ± SEM)	***n*** (% ± SEM)
BSA	67	64 (95.4 ± 2.8)	56 (78.5 ± 6.6)	51 (68.6 ± 8.9)	49 (67.1 ± 8.1)^a^
BSA-EV	52	48 (93.9 ± 6.1)	43 (84.2 ± 8.7)	41 (79.2 ± 9.8)	39 (73.1 ± 10.7)^a^
dFCS	86	80 (93.4 ± 2.6)	75 (86.6 ± 4.0)	67 (75.8 ± 5.7)	61 (69.2 ± 5.8)^a^
dFCS-EV	64	63 (97.9 ± 2.1)	60 (93.3 ± 4.1)	57 (91.2 ± 4.3)	54 (87.8 ± 5.7)^b^

^a,b^Values with different superscripts within a column are significantly different (*P* < 0.05)

#### Total cell number, mitochondrial activity and lipid contents

**Fig. 4:**
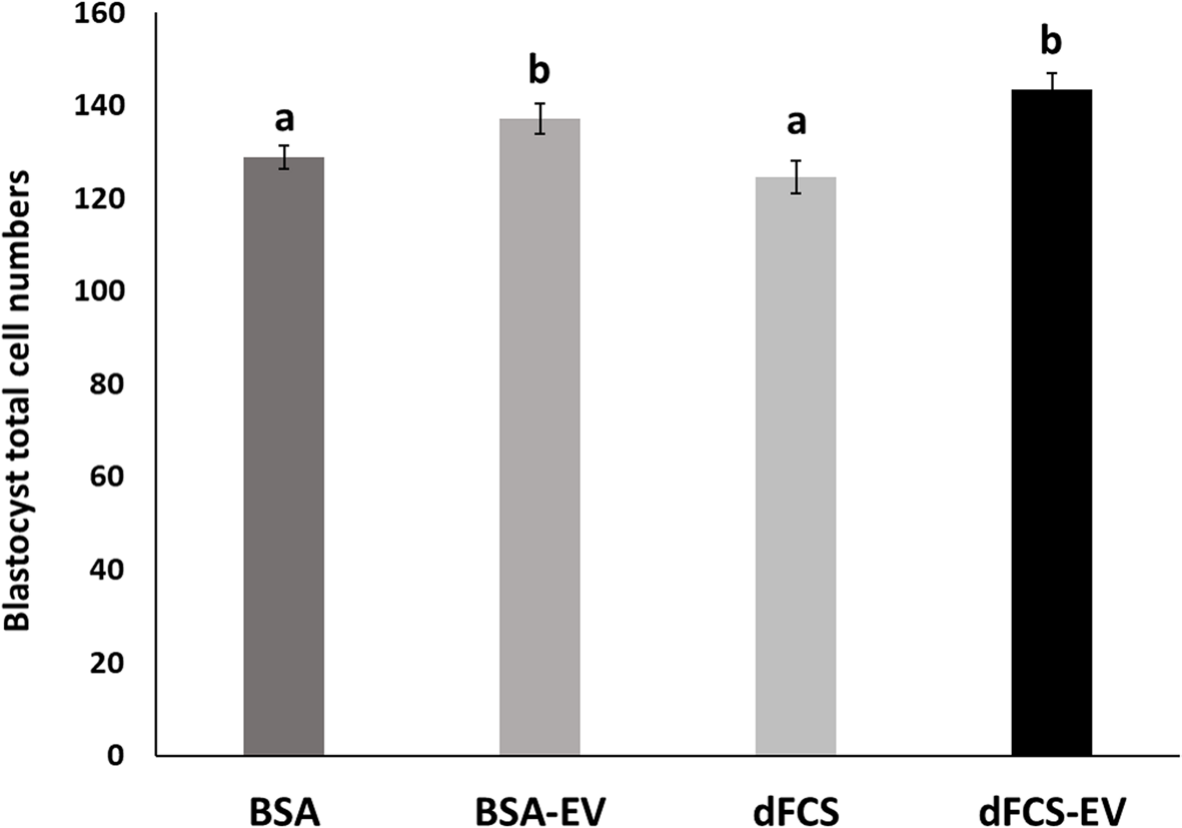
Total cell number in blastocysts IVC. Total cell number in blastocysts cultured in vitro with BSA or dFCS supplemented or not with OF-EVs (D1–4) and UF-EVs (D5–8) (BSA = 57, BSA-EV = 55, dFCS = 58, dFCS-EV = 56). Data are the means ± SEM. Different letters show statistically significant differences between treatments (*P* < 0.05)

As and additional parameter to assess possible effects of EVs on embryo quality, total cell numbers, mitochondrial activity and lipid contents in blastocysts were also evaluated. Results for total cell numbers in Fig. 4 show that although there was no variation in terms of blastocyst production by adding EVs to culture medium, the total nuclei in blastocysts showed differences among treatments. Both treatments with EVs did not differ between themselves (BSA-EV = 137.1 ± 3.2 and dFCS-EV = 143.5 ± 3.6, *P* > 0.05), but had increased cell numbers (*P* < 0.05) compared with their respective controls (128.8 ± 2.5 and 124.6 ± 3.5 for BSA and dFCS, respectively), which were also not different (*P* > 0.05). Therefore, the presence of EVs during embryo cultured showed a positive effect, irrespective of the type of protein supplementation used.

**Fig. 5:**
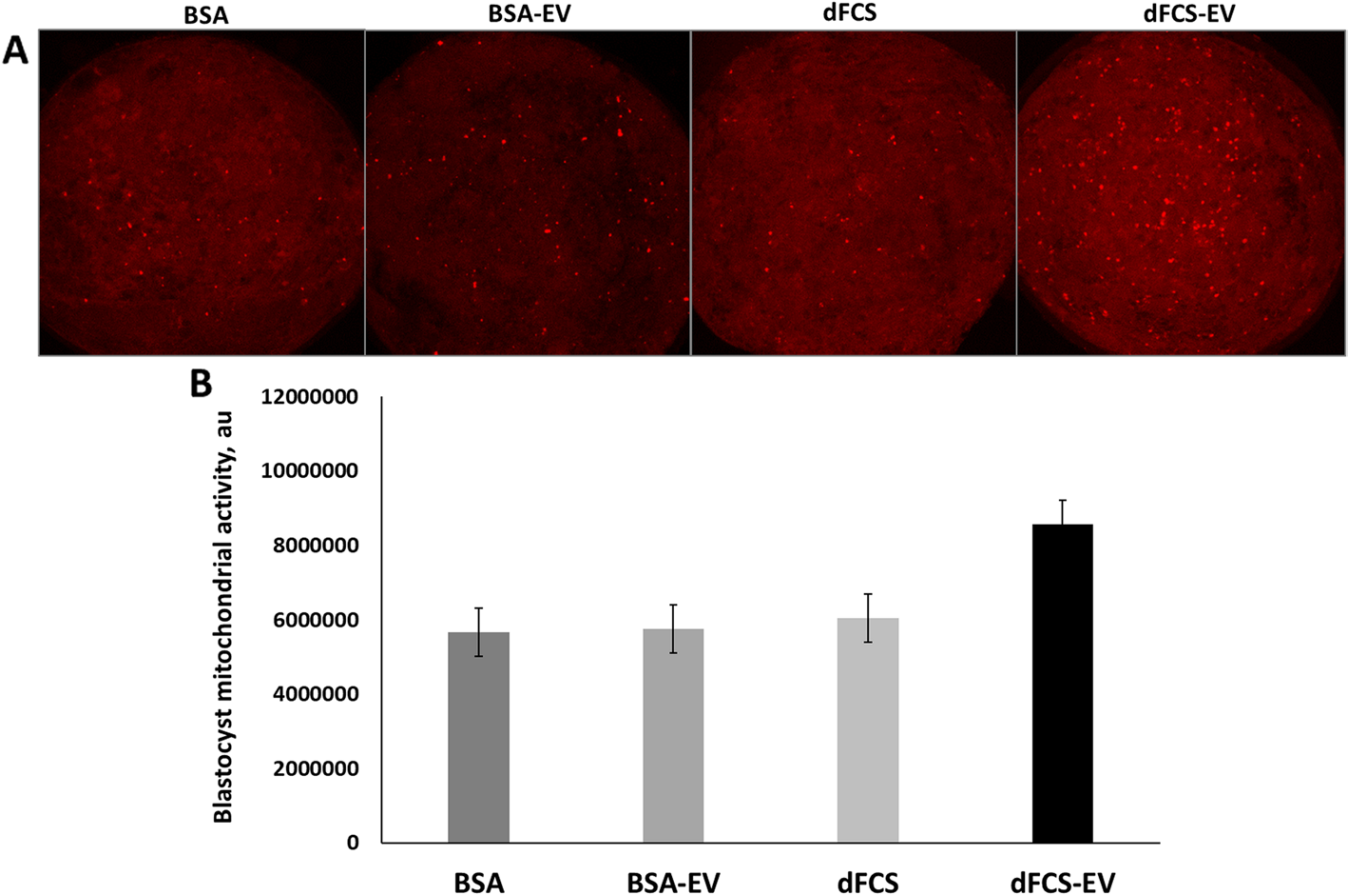
Representative fluorescence images of mitochondrial activity in bovine blastocysts. **A** Representative fluorescence images of mitochondrial activity in bovine blastocysts cultured in vitro in medium with BSA, BSA-EV, dFCS and dFCS-EV. Images captured on 63 × objective. **B** Quantification of mitochondria fluorescence intensity in arbitrary unites (au) in blastocysts cultured in vitro with BSA or dFCS supplemented or not with OF-EVs (D1–4) and UF-EVs (D5–8). (BSA = 29, BSA-EV = 29, dFCS = 33, dFCS-EV = 27). Data are the means ± SEM. (*P* > 0.05)

**Fig. 6:**
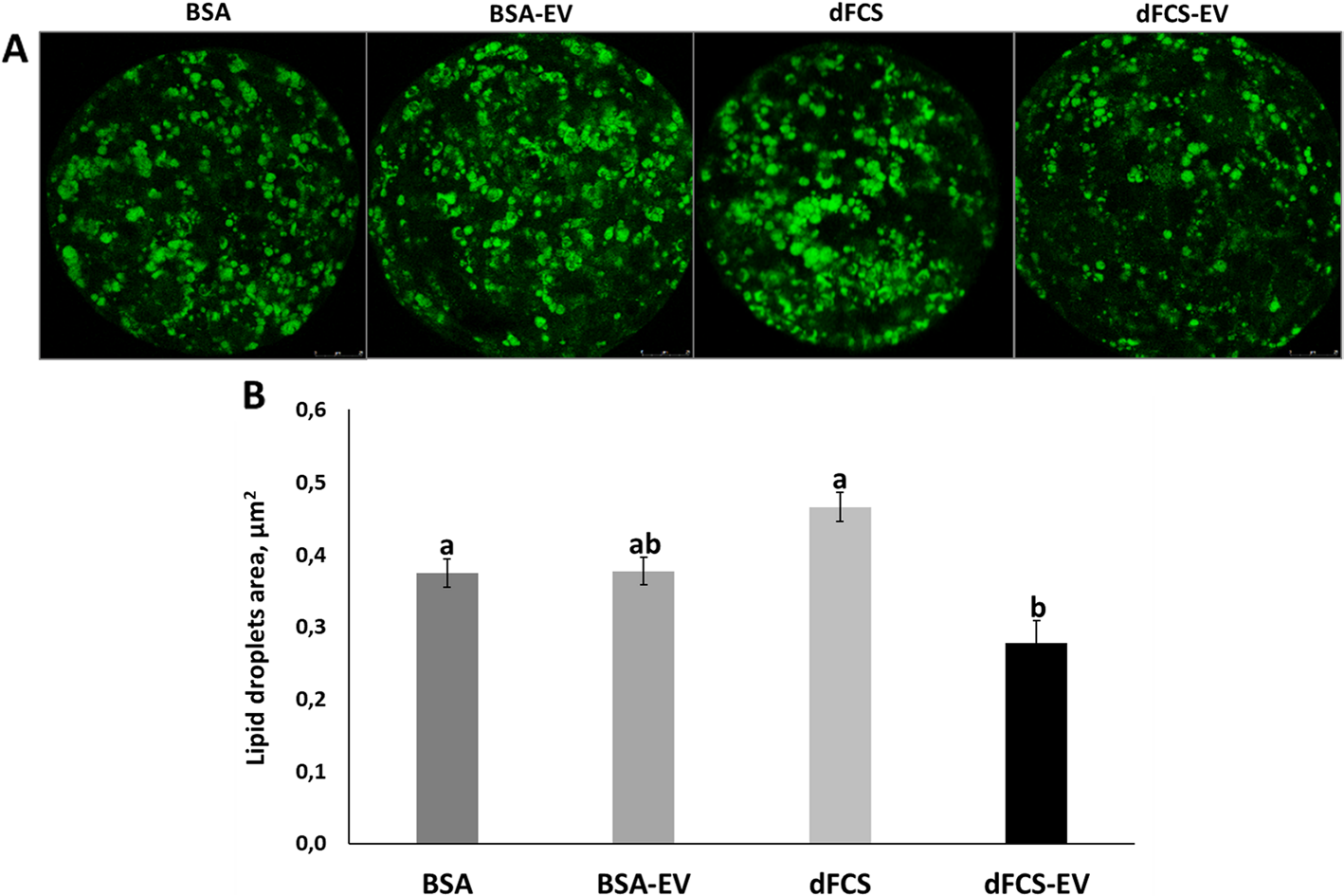
Representative fluorescence images of lipid droplets in bovine blastocysts. **A** Representative fluorescence images of lipid droplets in bovine blastocysts cultured in vitro in medium with BSA, BSA-EV, dFCS and dFCS-EV. Images captured on 63 × objective. **B** Quantification of total area of lipids in D7 - 8 blastocysts cultured in vitro with BSA or dFCS supplemented or not with OF-EVs (D1–4) and UF-EVs (D5–8) (BSA = 28, BSA-EV = 26, dFCS = 25, dFCS-EV = 29). Data are the means ± SEM. Different letters show statistically significant differences between treatments (*P* < 0.05)

Regarding mitochondrial activity (Fig. 5), EVs supplementation did not affect embryos in any treatment, nor did protein supplementation (*P* > 0.05). In respect of lipid contents (Fig. 6), the treatment with dFCS associated with EVs showed reduction (dFCS-EV = 0.277 ± 0.03 lipid droplet area in μm^2^, *P* < 0.05) compared with its control (dFCS = 0.466 ± 0.02 μm^2^) and to BSA (0.374 ± 0.02 μm^2^), but BSA-EV (0.377 ± 0.02 μm^2^) was not different from BSA or dFCS treatments (*P* > 0.05). Thus, in embryos cultured with dFCS, the presence of EVs caused a reduction in lipid contents, while in those cultured with BSA, the addition of EVs had no effect.

### Gene expression

**Fig. 7:**
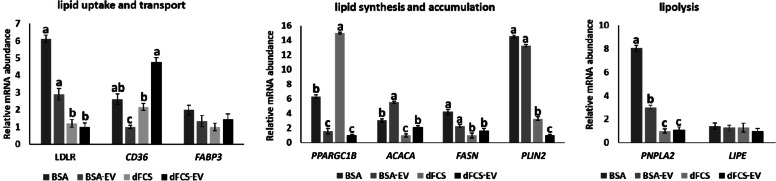
Relative mRNA abundance of lipid metabolism-related genes in blastocysts. Relative mRNA abundance of lipid metabolism-related genes in blastocysts cultured in vitro with BSA or dFCS only or supplemented with OF-s (D1–4) and UF-EVs (D5–8) (BSA, BSA-EV, dFCS and dFCS-EV). Bars represent the relative abundance of the transcripts analysed and normalized to *H2AFZ* and *ACTB* as housekeeping genes. The experimental treatments are represented by columns. Data are the means ± SEM. Different letters show statistically significant differences between treatments (*P* < 0.05)

Lipid contents in cells is influenced by different processes including lipolysis, lipogenesis, lipid uptake and transport. Therefore, we have analysed expression of genes related with these processes (Additional file 1: Table S1) to determine if they were affected by EVs. As seen in Fig. 7, analyses have shown that there was variation in transcripts abundance regarding the presence of EVs in six of the nine genes analysed. *PPARGC1B* was downregulated by EVs in both types of medium (*P* < 0.05), while *CD36* was upregulated in embryos cultured with EVs only in dFCS (*P* < 0.05). Most effects of EVs were observed in embryos cultured in BSA medium, where transcripts were downregulated (*LDLR*, *FASN* and *PNPLA2*), except for *ACACA*, which was upregulated (*P* < 0.05). For all these transcripts, except *ACACA*, there was significant interaction between EVs and type of protein source in medium (*P* < 0.05). *PLIN2* was only affected by type of protein in medium, and was downregulated in dFCS treatments (*P* < 0.05). *FABP3* and *LIPE* were not affected by any treatment (*P* > 0.05).

### Lipolysis proteins

**Fig. 8:**
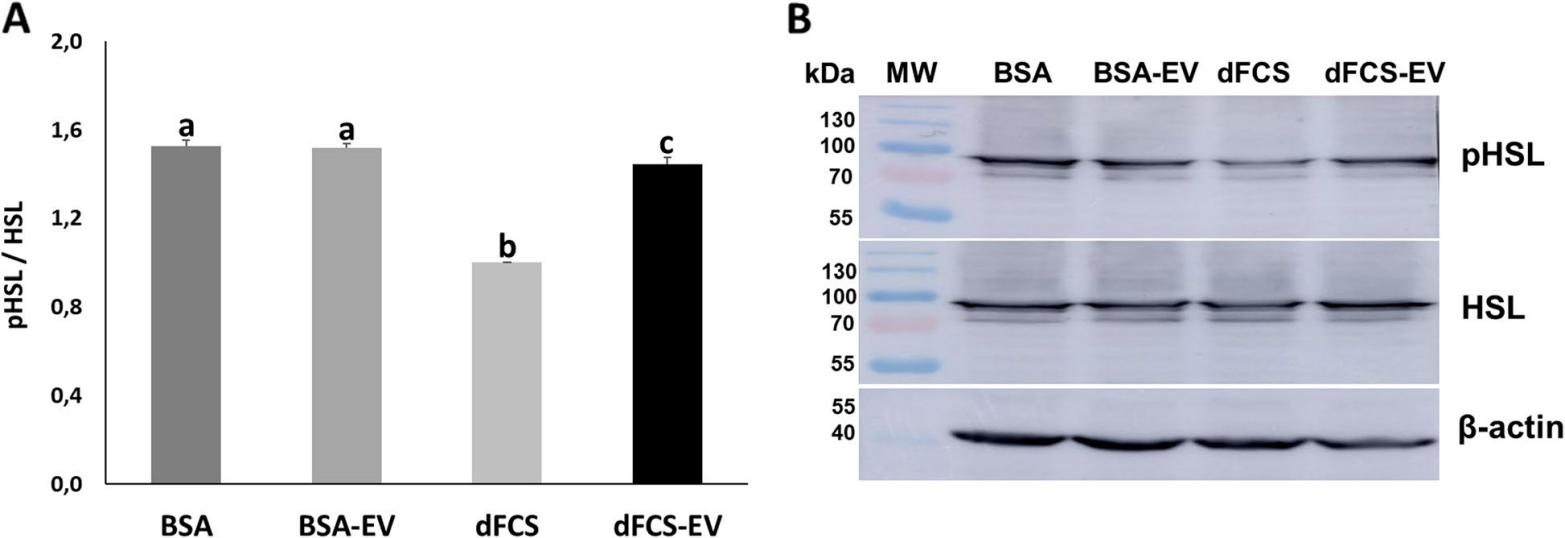
pHSL phosphorylation levels in bovine blastocysts. Effect of in vitro culture medium with BSA or dFCS supplemented or not with OF-EVs (D1–4) followed by UF-EVs (D5–8) on pHSL phosphorylation levels in bovine blastocysts. **A** pHSL phosphorylation level. Expression levels were normalized to the abundance of endogenous control actin. Phosphorylation level was expressed as pHSL/HSL. Data are expressed as means ± SEM. Different letters show statistically significant differences between treatments (*P* < 0.05). **B** Representative images of membranes treated for detection of lipolysis protein, total HSL and phosphorylated p-HSL, and β-actin. MW = molecular weight marker. Numbers indicate the MW. Expected MW: pHSL and HSL = 81 kDa and β-actin = 42 kDa

In the previous analyses we observed that several lipid metabolism transcripts for distinct metabolic processes were affected, and also lipid contents in embryos was reduced by EVs, which could be due to increased lipolysis. Therefore, we analysed the protein levels for total HSL (rate-limiting enzyme for lipolysis) and phosphorylated HSL (activated form of the enzyme), as an indication of activated lipolysis. Protein levels were determined by western blotting in D7–8 blastocysts cultured in medium with BSA, BSA-EV, dFCS and dFCS-EV. As observed in Fig. 8, results showed that HSL and pHSL were detected in all treatments and that HSL phosphorylation level was significantly lower (*P* < 0.05) in dFCS group compared with all treatments. The reduction was partially reverted by inclusion of EVs in dFCS medium, which increased pHSL levels relative to dFCS (*P* < 0.05).

### miRNA in EVs from OF and UF

**Fig. 9:**
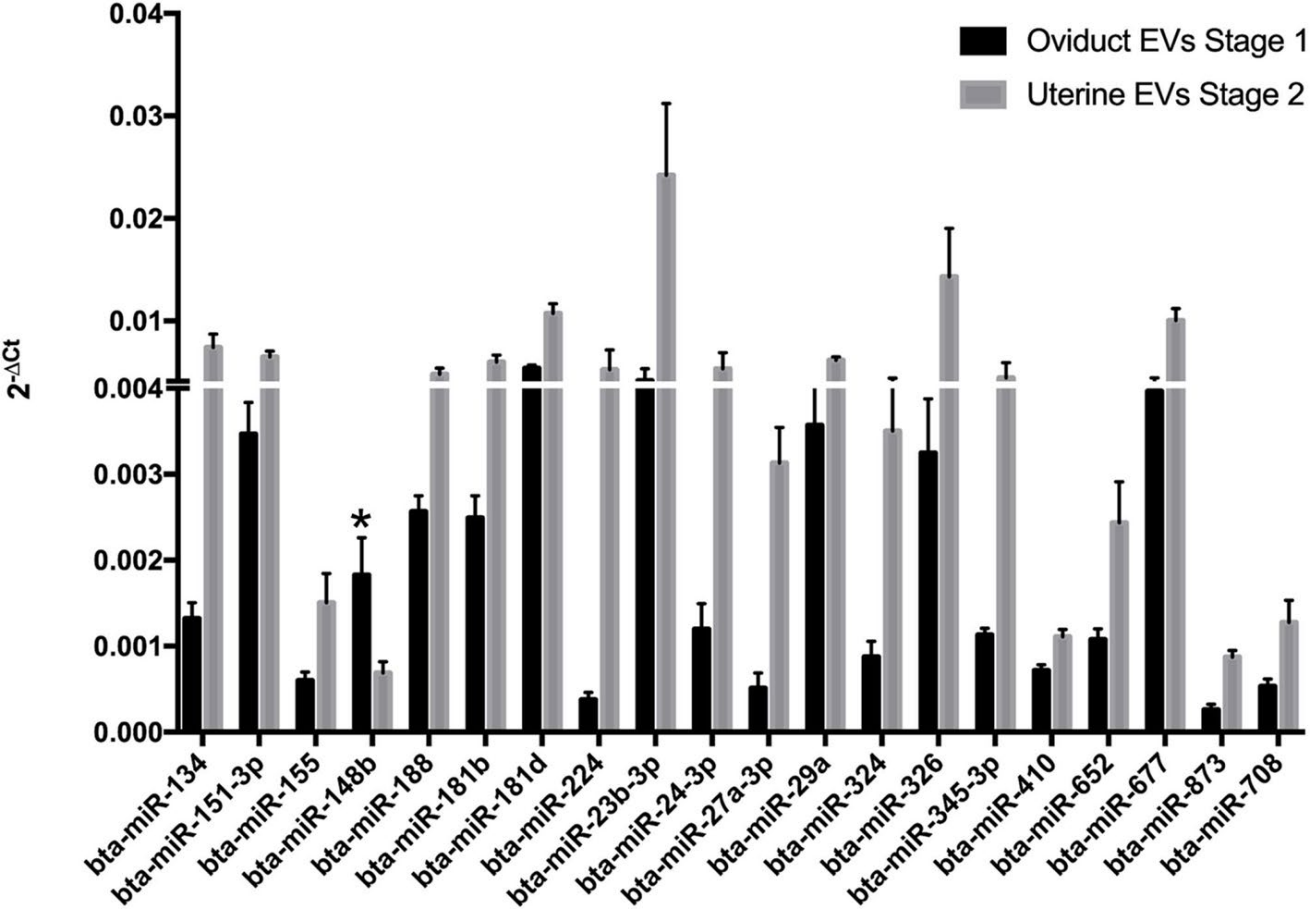
Relative abundance of miRNAs in EVs from OF and UF in bovine embryos. Relative abundance of miRNAs in EVs from OF and UF used in the in vitro culture of bovine embryos. From 20 differentially expressed miRNAs, 19 were upregulated in UF-EVs and one* in OF-EVs (*P* < 0.05)

As different parameters were affected by EVs and miRNAs are known to regulate cell functions and are transported by these vesicles, the miRNA contents of EVs in OF and UF were also analysed. Three hundred and thirty-three mature miRNAs were detected, from which the twenty most abundant in both fluids are listed in Additional file 2: Table S2. Most of them are common to EVs from both fluids, and the top four are the same (bta-miR-615, bta-miR-323, bta-miR-494 and bta-miR-631) and in a similar order of enrichment, with little variation. Only two were exclusive to OF (bta-miR-493 and bta-let-7a-5p) and two to UF (bta-miR-200c and bta-miR-1225-3p). When the abundance of miRNAs between the two EVs origins was compared, 20 were shown to be significantly differentially expressed (*P* < 0.05), and of these, 19 were more abundant in EVs from UF, while only one was more abundant in EVs from OF (bta-miR-148b, Fig. 9).

## Discussion

In the present study, we have used a sequential in vitro culture system, by supplementing embryo culture medium with EVs isolated from OF obtained at early luteal phase (Stage 1) and used during the first 4 d in culture, followed by use of EVs isolated from uteri obtained at mid-luteal phase (Stage 2) for the remaining days (up to day 9). This system intends to mimic the anatomical developmental sequence and the exposure of embryos to EVs from oviductal and uterine fluids, which would happen during early development in vivo, but not during IVC. The main findings were that although embryo development was not increased, embryo quality was improved in those cultured with EVs and that lipid metabolism-related genes, as well as an active lipase protein, are affected by their supplementation. Also, the protein source in the medium (BSA or dFCS in this study) may influence the functional effects of EVs on bovine embryos produced in vitro.

### EVs characterization

EVs isolated from OF and UF were characterised by NTA, TEM and WB, and presented the expected features by the three methods, confirming we have isolated EVs. EVs had the typical cup-shaped morphology, as previously described (bovine oviduct [36, 50] and uterus [38, 39]), and the marker proteins (HSP70 [50] and CD9 [36] in oviduct EVs and HSP70 [38] and CD9 [39] in uterus EVs). Reported concentrations for oviduct EVs were about 5 × 10^8^ to 1.0 × 10^9^ particles/mL [50] and 7.5 to 10.5 × 10^8^/mL [36], and sizes smaller than 200 nm [36]. EVs from Stage 2 uteri were not assessed for concentration, and only size was determined by TEM, between 50 and 150 nm [39]. In EVs isolated from bovine uteri at different days of pregnancy (17 to 22 d), Kusama et al. [38] also reported sizes of 50–150 nm determined by TEM and 109 to 137 nm by NTA. Particle concentrations were in the order of 10^6^ particles/mL. In the present study sizes were about similar to the others (≤ 200 nm), but concentrations were higher (~ 10^10^ particles/mL). These differences are probably due to distinct isolation methods, as [50, 36, 38] used two UC and [39] used precipitation solution, while we have used SEC combined with one UC. Isolation methods are known to affect particle concentration, purity and size [51].

### EVs uptake

Blastocysts cultured with labelled EVs from OF and UF presented fluorescent spots in blastomeres, showing that embryos internalize EVs from both origins and at both stages of development (from OF in D4 embryos and from UF in D7 blastocysts). Labelled EVs from OF, FF or derived from embryo-conditioned medium were shown to be uptaken by bovine blastocysts [37, 52, 53], but from UF has only been reported in D14 ovine concepti [54]. We have now demonstrated that bovine blastocysts also internalize UF-EVs, and that earlier stage embryos (8-cell) also uptake EVs. Pores in zona pellucida vary in number and diameter in bovine embryos; numbers increase while diameter slightly reduces (203 to 155 nm) from 8-cell to morula stage [55]. Average size of EVs isolated in this study (178 nm from OF and 151 nm from UF) match the pore sizes throughout development and can be uptaken by embryos. Therefore, both types of EVs could deliver their contents (proteins, mRNA, miRNA, among other molecules [56]) and affect embryos at different developmental stages.

### In vitro embryo development

EVs supplementation during embryo culture in vitro did not improve embryo development, as blastocyst rates were similar in dFCS and dFCS-EV treatments. Surprisingly, however, development was reduced when EVs were added to BSA containing medium. When culturing embryos with complete FCS and EVs isolated from BOECs conditioned medium, lack of effect of EVs have also been reported [28]. In contrast, positive effect of EVs on embryo development has been observed by Qiao et al. [39] when using EVs only from UF in medium with BSA from D4 and by Almiñana et al. [37] using EVs only from OF in medium with dFCS. Differences in culture conditions (only OF or only UF EVs vs. sequential use of OF and UF-EVs), type of embryo (cloned embryos in [39]), EVs isolation method and amount of EVs supplemented to culture medium, could account for the diverging results between studies. Regarding EVs in medium with BSA, Lopera-Vasquez et al. [36] and Banliat et al. [57] did not report any effect of EVs, contrary to our observations, but they both used different isolation methods. No other studies have directly compared EVs addition in culture media with different protein supplementation, so the reasons for diverging effects of EVs when using BSA or dFCS are unknown, but as mentioned earlier, protein supplement could affect EVs uptake, which could lead to different effects on cells.

### Embryo quality

#### Vitrification

When assessing embryo quality by blastocyst survival rates at 72 h after vitrification and warming, addition of EVs had no effects in medium with BSA, but in dFCS survival was increased (87.8% vs. 69.2%, respectively), indicating a positive effect of EVs in this treatment. EVs from BOECs conditioned-medium increased survival rates compared with complete serum (50% vs. 20%) [28]. However, EVs were added in medium devoid of any protein source, so the improved cryotolerance could be in fact due to the exclusion of serum, which was shown to increase cryotolerance (56% vs. 26%, [29]). Nonetheless, a positive effect of oviduct EVs has been described when they were isolated from the isthmus of the oviduct (80% vs. 51%) [36]. Besides, the sequential use of low concentrations of OF followed by UF also improved cryotolerance compared with FCS [30]. This could be related with the EVs in fluids, as observed in the current study using a similar model, but with EVs.

#### Total cell numbers

Blastocyst quality was also assessed by other parameters including total cell numbers and a positive effect was observed. Similar outcome was reported for EVs from BOECs-conditioned medium with BSA [28], from UF in medium with BSA [39], and from OF in medium with dFCS [37]. EVs isolated from medium conditioned by cultured embryos also show this same effect [54, 58].

Such effect would be due to a stimulus to cell proliferation as reported by Hung et al. [59], who observed that EVs from follicular fluid increased granulosa cells proliferation in vitro. Besides, transcripts for genes related with cell cycle regulation were detected in OF EVs [50], and could be transferred to embryos after uptake, stimulating cell division. Such possibility seems plausible, as horizontal transfer of mRNAs and proteins between bovine granulosa cells were shown to occur through EVs, and phenotypical changes in target cells, including increased proliferation, have also been reported [60].

#### Mitochondrial activity

Regarding mitochondrial activity in blastocysts, no effects were detected. These results suggest that EVs, under the conditions studied, do not influence mitochondrial activity. There are no studies assessing the influence of EVs on mitochondrial activity of bovine embryos, although a positive effect of EVs has been reported in bovine granulosa cells [60]. However, these are different cell types under distinct culture conditions to be compared, and more studies should be performed to investigate a possible effect of EVs from reproductive fluids on mitochondrial function in cultured embryos.

#### Lipid contents

When analysing lipid contents, the dFCS-EV treatment showed reduction, suggesting effects of EVs when embryos are cultured with serum, but not with BSA. Substitution of serum for BSA in embryo culture medium has been shown to decrease lipids [61], and apparently the supplementation of EVs did not result in an additional decrease. In the present study, we have used dFCS, which was shown to improve embryo quality and cryotolerance [28], as also observed with the use of BSA [30], suggesting a possible reduction in lipids. In fact, dFCS lipid contents were not different from BSA. On the other hand, EVs were capable of causing a significant reduction in embryo lipids compared with dFCS alone, which was even lower that BSA alone. Almiñana et al. [50] have detected several transcripts and miRNAs related with lipid metabolism. Therefore, EVs could affect embryo lipid metabolism through modifications on mRNA and/or protein expression. Taken together, the observations for total cell number and lipid contents, as well as cryotolerance, indicate that EVs improve some crucial parameters related with embryo quality.

### Expression of lipid-metabolism genes

The presence of EVs affected six of nine analysed transcripts (*LDLR, PPARGC1B*, *ACACA*, *CD36*, *FASN,* and *PNPLA2*), with varied patterns of expression, and except for *ACACA*, there was interaction with the protein source in medium. Most transcripts were decreased by addition of EVs (*PARGC1B* in BSA and dFCS and *LDLR, FASN,* and *PNPLA2* in BSA) and two were increased (*ACACA* in BSA and *CD36* in dFCS). The most common outcome of miRNAs on mRNA levels is a decrease by degradation of the targeted transcript [62], however, increased transcription mays also be induced by miRNAs [63, 64], so some of the expression effects could be due to miRNAs in EVs. On the other hand, increased transcripts in target cells may be a result of direct transfer of mRNA, and this could be the case for *ACACA* and *CD36*, as their transcripts were identified in OF-EVs [50]. mRNAs for *PPARGC1B*, *PNPLA2*, *LDLR*, *FASN* and *PLIN2* were also detected in OF-EVs by Almiñana et al. [50], but they were decreased in embryos (except *PLIN2*, reduced by dFCS but not EVs), so transfer of mRNAs would not explain this outcome. Bauersachs et al. [65] have analysed mRNA and miRNAs in OF-EVs and compared with the transcriptome of embryos cultured with them. Authors concluded that EVs regulate expression in embryos by different mechanisms including increased delivery of mRNAs, translation of delivered mRNAs into proteins that then modulate expression or by miRNAs that downregulate mRNAs or modify expression in other ways. Whether miRNAs and/or mRNAs in EVs used in this study are influencing these transcripts cannot be discriminated, and warrant further studies.

Another observation regarding effects of EVs, was that some transcripts were affected differently depending on the protein source in culture medium. Most transcripts were modulated by EVs in BSA treatment (*LDLR, ACACA*, *FASN,* and *PNPLA2*), and only one in dFCS (*CD36*). *PPARGC1B* was the only transcript downregulated by EVs irrespective of protein source in medium. Reasons for this outcome are unknown. BSA does not contain EVs [53], but serum, although depleted of its EVs, still has some left as the standard depletion process (ultracentrifugation) is not complete [66, 67]. Thus, some EVs still in dFCS could lead to a different effect than that of medium with BSA. Also, if culture medium supplementation can somehow affect uptake of EVs as in certain cell lines [68], this would affect cargo delivery and the response of target cell.

Taken together, EVs were shown to affect transcripts levels of lipid metabolism-related genes involved in lipogenesis (*PPARGC1B*, *FASN* and *ACACA*), lipolysis (*PNPLA2*) and lipid uptake (*LDLR* and *CD36*), therefore EVs from OF and/or UF can influence lipid metabolism of embryos. Also, type of protein source in culture medium may interfere with the effect of these EVs in vitro.

### Lipid metabolism proteins

HSL is an essential enzyme in lipolysis and is activated when phosphorylated [69]. HSL protein and its phosphorylated form (pHSL) were detected in all treatments, showing that the enzyme is present and functional in bovine embryos. This protein had only been detected in bovine oocytes and cumulus cells [70, 71], so this is the first confirmation of its presence also in embryos, indicating its certain participation in embryonic lipid metabolism. HSL phosphorylation level was lower in dFCS, suggesting this treatment would have lower lipolysis rates, that could be related with the higher amount of lipids found in this treatment. Addition of EVs in medium with dFCS had a positive effect on the stimulation of lipolysis and could counteract the effect of serum. Recently, Zhuan et al. [72] have shown in porcine oocytes that inhibition of HSL activity resulted in increased lipids in oocytes. This is in accordance with our observation that lower active HSL corresponded with higher lipid amounts in dFCS blastocysts, and addition of EVs restored active HSL levels, in line with the lower lipid contents in dFCS-EVs treatment.

Interestingly, transcript levels for HSL did not vary with any treatment, further supporting the idea that transcript levels do not correlate to lipases activities [73], and that detection of the active form of the enzyme can be a better indicator of effects on lipid metabolism. The mechanism by which EVs could affect activity of HSL is unknown, but EVs have been shown to participate in the regulation of cell metabolism by multiple pathways [74].

### miRNA in EVs used for IVC

As EVs carry different types of cargo, including miRNAs, which modulate target-cell function through effects on the expression of transcripts and proteins [62–64]. Therefore, we also looked at their content in the EVs used in our culture system. From the twenty most abundant miRNAs in EVs from OF and UF, bta-miR-200b was also detected by Almiñana et al. [50] in bovine EVs from OF. When comparing miRNA contents in EVs from OF and UF, two of the differentially expressed (bta-miR-151-3p and bta-miR-24-3p), were also reported in EVs from OF [50], in agreement with present observations. These two miRNAs are involved in the control of expression of *FASN* and *ACACA* [50], and both were influenced by the EVs supplementation in the culture medium, but with different outcomes. *ACACA* was upregulated and *FASN* downregulated by EVs. Both or either one of these outcomes could due to one or both of these miRNAs, as these molecules may either induce transcription (*ACACA*) [63, 64] or transcript degradation (*FASN*) [67].

Some of the miRNA detected in EVs from OF and UF samples, such as bta-miR-181, bta-let-7a, bta-let-7b, bta-miR29a, bta-miR-151 and bta-miR-494 have been related with implantation in mice [75, 76], sheep [77] and humans [78]. From the 20 differentially expressed, almost all of them were more abundant in EVs from UF, suggesting that these miRNAs could be involved with the arrival of the embryo to the uterus. The only miRNA that was lower in EVs from UF compared with those from OF (bta-miR-148b) was reported to be related to the suppression of proliferation, migration, and invasion in human tumour cells [79], suggesting that its reduction in the uterus would allow for an increase in these cell activities. Also, interferon-τ secretion by embryonic trophoblastic cells, lowers the expression of this miRNA to reduce the inflammatory response in bovine endometrial epithelial cells [80]. Therefore, the reduction of bta-miR-148b in the uterus compared with the oviduct, could be related with favouring the implantation process. As nearly all differentially expressed miRNAs were upregulated in the uterine EVs, this is probably a reflection of the highly complex function of the uterus in regulating embryo development and the process of implantation and placental formation. Studies on uterine EVs and miRNAs in bovine have mostly addressed the peri-implantation period (D16–22, [38, 81–84]) and have shown the importance of embryo-maternal communication through EVs during this period [85]. However, the cross-talk between the embryo and uterine epithelium is quite intense also at early stages of development (D5–8 as in our model [86];), and EVs and miRNAs may probably participate in this communication. Interestingly, one of the upregulated miRNAs in UF-EVs was bta-miR-155, which was shown to be more expressed in IVD embryos between D7–9 of development compared with IVP [87]. This miRNA could be indicative of better quality of embryos as it may be transferred or induced by uterine EVs.

As there are no studies comparing EVs from OF and UF and especially regarding the miRNAs in EVs from bovine UF, the roles of these miRNAs for early embryo development remain to be investigated. Although miRNAs in EVs can regulate functions in target-cell [88], it is also possible that some of the effects reported in this study were due to the other constituents of EVs cargo. Bauersachs et al. [65] reported that both mRNA and miRNA in OF EVs regulate the transcriptome of embryos. Lipidome in EVs is less studied [89], but recently Banliat et al. [57] observed changes in embryonic lipids profile after exposure to OF EVs. Therefore, it must be considered that different cargoes (mRNA, proteins, bioactive lipids, among others), may also be involved in effects caused by EVs during IVC.

## Conclusions

Although EVs do not affect embryo yield, they improve quality parameters, and it would be interesting to assess the ultimate indicator of embryo quality, which is in vivo development to determine long term effects of these vesicles. Nevertheless, the in vitro study has shown that EVs lead to positive outcomes related with increased cell number, reduced lipid contents and increased post-vitrification survival (dFCS-EVs). Such observations are at least partly given by effects on expression of lipid metabolism related genes, which are modulated by mRNAs, miRNAs and/or proteins contained in these EVs, besides an increase in the active form of HSL, suggesting activated lipolysis. Further studies are needed to understand which cargo may be affecting which cellular functions and how they may be leading to the observed results.

## Supplementary Information

**Additional file 1: Table S1.** Details of primers used for reverse transcription–quantitative polymerase chain reaction.

**Additional file 2: Table S2.** Top 20 miRNA in EVs from OF and UF.

## Data Availability

The raw data supporting the conclusion of this article will be made available by the authors, without undue reservation, to any qualified researcher. In addition, all generated or analysed data derived from this study are included in this published article and its Supplementary data files.

## Abbreviations

IVC: In vitro culture
IVP: In vitro production
EVs: Extracellular vesicles
OF: Oviductal fluid
SOF: Synthetic oviductal fluid
UF: Uterine fluid
dFCS: EVs-depleted fetal calf serum
FCS: Fetal calf serum
BSA: Bovine serum albumin
miRNAs: microRNAs
NTA: Nanoparticle tracking analysis
TEM: Transmission electron microscopy
LDLR: Low density lipoprotein receptor
ACACA: Acetyl-CoA carboxylase alpha, previous name ACC
FASN: Fatty acid synthase, alias FAS
PLIN2: Perilipin 2
PNPLA2: Patatin like phospholipase domain containing 2, alias ATGL adipose triglyceride lipase
PPARGC1B: PPARG coactivator 1 beta
FAPBP3: Fatty acid binding protein 3
LIPE: Lipase E, hormone sensitive type, alias HSL Hormone sensitive lipase
