# Supplementary material for: Extracellular vesicles from oviductal and uterine fluids supplementation in sequential in vitro culture improves bovine embryo quality
Source: J Anim Sci Biotechnol. 2022 Oct 25;13:116. doi: 10.1186/s40104-022-00763-7 (PMC9594899; doi:10.1186/s40104-022-00763-7)
Supplement: Supplementary file 1 — Additional file 1: Table S1. Details of primers used for reverse transcription–quantitative polymerase chain reaction. [file 40104_2022_763_MOESM1_ESM.docx]

**Table S1**  Details of primers used for reverse transcription–quantitative polymerase chain reaction

| Gene symbol (name) | Gene primer sequence (5’ → 3’) | Fragment size, bp | GenBank accession number |
| --- | --- | --- | --- |
| **Lipolysis** |  |  |  |
| *PNPLA2* (patatin like phospholipase domain containing 2, alias *ATGL* adipose triglyceride lipase) | TGCCAGTACCTGATGATACGC  CCTCCCATTTGGCCAGTACAT | 192 | XM_005227323.3 |
| *LIPE* (lipase E, hormone sensitive type, alias *HSL* hormone sensitive lipase) | CGGGAGGCTCTTCTTTGAGG  TGGAGATGGTCTGCAGGAAC | 146 | NM_001080220.1 |
| **Lipogenesis** |  |  |  |
| *ACACA* (acetyl-CoA carboxylase alpha, previous name *ACC*) | AAGCAATGGATGAACCTTCTTC  GATGCCCAAGTCAGAGAGC | 196 | FN185963.1 |
| *FASN* (fatty acid synthase, alias *FAS*) | CTGGCGGCATCTACATCTCAAG  ACCAGCTAGCACCACCTTCAT | 473 | NM_001012669.1 |
| *PPARGC1B* (PPARG coactivator 1 beta) | GCCTCCTTCAGTAAGCTGTCAA  GGCCCCGCTATACTGACTATGA | 101 | XM_005209630 |
| *PLIN2* (perilipin 2) | ACAACACACCCCTCAACTGG  CTGCCTGCCTACTTCAGACC | 2111 | NM_173980.2 |
| **Lipid uptake/transport** |  |  |  |
| *LDLR* (low density lipoprotein receptor) | CAAAACCCCGATCATCCCCA  TCGACCCTGAACTGGAAACG | 194 | NM_001166530 |
| *CD36* (cluster of differentiation 36, alias *FAT* fatty acid translocase) | GCTGCAGGTCAACATGCTGGTC  CGATTTCTACCAGGCCCAGGAG | 182 | NM_001278621.1 |
| *FABP3* (fatty acid binding protein 3) | TTGTGCGGGAGATGGTTGA  TGCCGAGTCCAGGAGTAGCC | 147 | NM_174313.2 |
| **Housekeeping** |  |  |  |
| *H2AZ1* (H2A.Z variant histone 1, previous name *H2AFZ*) | AGGACGACTAGCCATGGACGTGTG  CCACCACCAGCAATTGTAGCCTTG | 212 | NM_016750 |
| *ACTB* (actin beta) | GAGAAGCTCTGCTACGTCG  CCAGACAGCACCGTGTTGG | 264 | AF191490.1 |
