# Supplementary material for: Extracellular vesicles from oviductal and uterine fluids supplementation in sequential in vitro culture improves bovine embryo quality
Source: J Anim Sci Biotechnol. 2022 Oct 25;13:116. doi: 10.1186/s40104-022-00763-7 (PMC9594899; doi:10.1186/s40104-022-00763-7)
Supplement: Supplementary file 2 — Additional file 2: Table S2. Top 20 miRNA in EVs from OF and UF. [file 40104_2022_763_MOESM2_ESM.docx]

**Table S2** List of 20 most abundant miRNAs in EVs from OF and UF used in in vitro culture of bovine embryos

| **OFS1** | **UFS2** |
| --- | --- |
| bta-miR-615 | bta-miR-615 |
| bta-miR-323 | bta-miR-323 |
| bta-miR-494 | bta-miR-631 |
| bta-miR-631 | bta-miR-494 |
| bta-miR-1224 | bta-miR-1246 |
| bta-miR-1307 | bta-miR-1224 |
| bta-miR-1246 | bta-miR-1307 |
| bta-miR-1260b | bta-miR-1343-5p |
| bta-miR-1343-5p | bta-miR-1260b |
| bta-let-7b | bta-miR-149-3p |
| bta-let-7c | bta-miR-574 |
| bta-miR-425-3p | bta-miR-425-3p |
| bta-miR-149-3p | bta-let-7b |
| bta-miR-940 | bta-let-7c |
| bta-let-7e | bta-miR-940 |
| bta-miR-486 | bta-miR-200b |
| **bta-miR-493** | bta-let-7e |
| **bta-let-7a-5p** | bta-miR-486 |
| bta-miR-574 | **bta-miR-200c** |
| bta-miR-200b | **bta-miR-1225-3p** |

In bold, exclusive miRNA in EVs for each fluid
